# Infant weight gain and DXA-measured adolescent adiposity: data from the Japan Kids Body-composition Study

**DOI:** 10.1186/s40101-021-00261-1

**Published:** 2021-08-27

**Authors:** Yuki Fujita, Katsuyasu Kouda, Kumiko Ohara, Harunobu Nakamura, Chikako Nakama, Toshimasa Nishiyama, Masayuki Iki

**Affiliations:** 1 Department of Public Health, Kindai University Faculty of Medicine, Osaka-Sayama, Osaka, 589-8511 Japan; 2 Department of Hygiene and Public Health, Kansai Medical University, Hirakata, Osaka, 573-1010 Japan; 3 Department of Human Development, Graduate School of Human Development and Environment, Kobe University, Kobe, 657-8501 Japan

**Keywords:** Body fat mass, Birthweight, Child, Rapid weight gain

## Abstract

**Background:**

Rapid weight gain in early life is associated with adiposity later in life. However, there is limited information on the association between weight gain and body fat mass measured using precise methods. This study aimed to investigate whether weight gain is associated with body fat mass measured by dual-energy X-ray absorptiometry (DXA) in adolescents.

**Methods:**

Participants of this retrospective cohort study were 423 adolescents born at full-term who were enrolled in the Japan Kids Body-composition Study. Anthropometric measurements related to pregnancy, delivery, and child health were obtained from the Japanese Maternal and Child Health Handbook. Fat mass in adolescents was measured with a DXA scanner. Weight gain was defined as the change in body weight from birth to age 1.5 years. Associations between birthweight and fat mass, and between weight gain and fat mass, were evaluated using multiple regression analysis.

**Results:**

There was a significant positive association between weight gain from birth to age 1.5 years and fat mass in adolescents (boys: standardized regression coefficient (*β*) = 0.253, *p* < 0.01; girls: *β* = 0.246, *p* < 0.01), but not between birthweight standardized for gestational age and fat mass.

**Conclusion:**

Children with a greater change in weight from birth to age 1.5 years tended to have increased fat mass in adolescence. Weight gain in early life has a greater impact on fat mass in adolescents than birthweight.

## Background

Being overweight or obese is a major risk factor for cardiometabolic disease [1]. Childhood obesity tends to persist into adulthood and is associated with cardiovascular diseases and mortality [2]. Some studies have reported that preterm neonates with low weight become obese in childhood [3, 4]. According to a recent meta-analysis, preterm neonates with low weight tend to experience rapid weight gain (RWG, defined as a large weight gain velocity in early life) and subsequent childhood obesity [4–7]. Full-term neonates with high weight also tend to become obese children at school age, as compared with full-term neonates with normal weight [8–10]. These previous reports suggest that there are several risk factors for childhood obesity, such as gestational age, birthweight, and RWG in early life. For full-term neonates, we previously reported that adolescents who experienced RWG in early life were likely to have a large body mass [7].

Since overweight and obesity reflect abnormal or excessive fat accumulation, it is important to evaluate body fat mass, rather than body mass, in adolescents. The Generation R Study in Rotterdam found that children with RWG tended to have a higher skinfold thickness (subcutaneous fat thickness) at pre-school age, as compared to children without RWG [9]. However, there is limited information in the literature regarding the association between RWG and body fat mass measured using precise methods. Dual-energy X-ray absorptiometry (DXA) can be used to determine the bone and soft tissue composition of the entire body, including fat mass, and the precision of DXA measurements has been reported to be excellent [11]. Accordingly, the present study aimed to investigate whether RWG in early years is associated with DXA-measured body fat mass in adolescents.

## Methods

### Study design and participants

The present study was a population-based retrospective cohort study which targeted adolescents enrolled in the Japan Kids Body-composition Study (JKB Study) [12]. The source population was 800 children aged 13–14 years who were registered at Shiokawa Junior High School in 2010, 2013, and 2016, and at Sekishi Junior High School in 2013 and 2014 in Hamamatsu, Japan. Of the source population, 532 students participated in the present study. The present analyses included 423 children (205 boys), after excluding 71 children with incomplete data, 36 children who were born at < 37 weeks gestation, and 2 children who were born at > 41 weeks gestation. The parent or guardian of each child read an information letter regarding study procedures, including the radiation dose from DXA measurements, and provided their informed consent before students participated. Students were also allowed to decline participation on their own accord, ever during the examination. The present study was approved by the Research Ethics Committee of the Kindai University Faculty of Medicine.

### Exposure variables and covariates

Anthropometric measurements related to pregnancy, delivery, and child health were obtained from the Japanese Maternal and Child Health (MCH) Handbook. The MCH Handbook consists of records of pregnancy, delivery, and child development and healthcare [13]. We obtained from the MCH Handbook information on the length/height and bodyweight of infants, as well as gestational age and maternal age at delivery. Data on pubic hair appearance and sedentary behavior were obtained from a self-administered questionnaire. Participants were asked to answer the question, “How much time do you spend doing sedentary activities (e.g., playing video games, using a computer or mobile phone, and watching TV) in a day?”, and selected one category from the following choices: “less than 1 h,” “from 1 h to less than 2 h,” “from 2 h to less than 3 h,” “from 3 h to less than 4 h,” “from 4 h to less than 5 h,” and “5 h and more”. Since the numbers of participants who selected “less than 1 h,” “from 4 h to less than 5 h,” and “5 h and more” were low, the other three categories were used in the analysis. Small for gestational age (SGA), appropriate for gestational age (AGA), and large for gestational age (LGA) categories are commonly defined as a weight below the 10th percentile, between the 10th and 90th percentiles, and greater than the 90th percentile, respectively, for gestational age and sex, as derived from Japanese neonatal anthropometric charts [14]. Body weight *z*-score (also referred to as the weight standard deviation (SD) score) independent of sex was calculated as [(measured body weight − reference mean body weight)/reference bodyweight SD], using Japanese growth charts from national data as the reference [15]. Weight gain was defined as a change in body weight *z*-score from birth to age 1.5 years. RWG was defined as a change of greater than 0.67 in body weight *z*-score from birth to age 1.5 years [16].

### Outcome variables

Bodyweight, height, and waist circumference (WC) at age 13–14 years were measured in light clothing and without shoes. Height was measured to the nearest 0.1 cm and weight was measured to the nearest 0.1 kg. Body mass index (BMI) (kg/m^2^) was calculated as body weight (kg) divided by height squared (m^2^). The cut-off point for overweight was defined as BMI 22.27 kg/m^2^ for boys and 22.98 kg/m^2^ for girls, in accordance with the International Obesity Task Force [17]. Body composition, corresponding to body fat mass and lean mass, was measured by a single DXA scanner (QDR-4500A, Hologic Inc., Bedford, MA, USA) in a mobile test room. Children wore light clothing without metal objects while undergoing whole-body scanning. An experienced radiological technologist performed all scans and scan analyses. Quality control of the DXA scanner was performed using the Step Phantom scan throughout the surveys [18]. Body fat mass (kg) was measured, and body fat percentage was calculated by dividing body fat mass by body mass. Height-normalized index of body fat mass (fat mass index: FMI, kg/m^2^) was calculated as whole-body fat mass (kg) divided by height squared (m^2^).

### Statistical methods

Statistical analysis was performed using SAS software for Windows, version 9.4 (SAS Institute Japan Ltd., Tokyo, Japan). Statistical significance was set at an alpha of 0.05 for all statistical tests. One-way analysis of variance (ANOVA) was used to compare anthropometric characteristics of mothers and their children according to birthweight for gestational age categories (SGA, AGA, and LGA; hereafter, “birthweight categories”). The unpaired t-test was used to determine whether the means of continuous variables in the two weight gain groups (non-RWG and RWG) significantly differed. The chi-square test was used to estimate independent proportions of sedentary behavior, pubic hair appearance, and overweight status according to birthweight categories and weight gain. For the ANOVA, t-test, and chi-square test, variables with a significant difference (*P* < 0.1) among birthweight categories, or between weight gain groups, were selected as potential confounding factors in analyses of the association between exposure and outcome. Adjusted means for BMI, WC, and fat mass were obtained as least square means from the analysis of covariance. Additionally, multiple regression analysis was used to test whether birthweight categories and weight gain in early life significantly predicted FMI in adolescence. Both birthweight categories and weight gain in early life were included in the multiple regression model.

## Results

Table 1 shows the mean values of anthropometric and body composition indices according to birthweight categories (i.e., SGA, AGA, and LGA). Gestational age in boys and maternal age in girls differed among birthweight categories. Change in weight z-score from age 0 to 1.5 years was larger in the SGA group, and smaller in the LGA group, compared to the other two groups for both boys and girls. FMI was significantly associated with birthweight categories in girls but not in boys.

**Table 1:** Characteristics of participants stratified by sex and birthweight for gestational age categories

	Boys				Girls			
	Birthweight for gestational age categories				Birthweight for gestational age categories			
	SGA (*N* = 10)	AGA (*N* = 151)	LGA (*N* = 44)	*p* value	SGA (*N* = 10)	AGA (*N* = 166)	LGA (*N* = 42)	*p* value
Maternal characteristics at pregnancy								
Age, years	26.6 ± 1.4	29.7 ± 0.4	29.3 ± 0.7	0.11	28.1 ± 1.6	28.5 ± 0.4	30.6 ± 0.8	0.05
Gestational age, weeks	39.9 ± 0.3	39.0 ± 0.1	38.9 ± 0.2	0.02	38.5 ± 0.3	39.0 ± 0.1	39.1 ± 0.2	0.26
Characteristics of the child at birth								
Length *z*-score	− 0.84 ± 0.24	0.06 ± 0.06	0.79 ± 0.12	< 0.01	− 1.5 ± 0.2	0.1 ± 0.1	0.9 ± 0.1	< 0.01
Weight *z*-score	− 1.36 ± 0.20	− 0.02 ± 0.05	1.57 ± 0.09	< 0.01	− 1.9 ± 0.2	− 0.2 ± 0.0	1.2 ± 0.1	< 0.01
Characteristics of the child at age 1.5 years								
Height *z*-score	− 0.23 ± 0.28	0.36 ± 0.07	0.87 ± 0.13	< 0.01	− 0.1 ± 0.3	0.1 ± 0.1	0.7 ± 0.2	< 0.01
Weight *z*-score	− 0.37 ± 0.28	0.25 ± 0.07	1.09 ± 0.13	< 0.01	− 0.2 ± 0.3	0.2 ± 0.1	1.1 ± 0.1	< 0.01
Change in weight *z*-score from 0 to 1.5 years	0.99 ± 0.31	0.27 ± 0.08	− 0.48 ± 0.15	< 0.01	1.7 ± 0.3	0.4 ± 0.1	0.0 ± 0.2	< 0.01
Characteristics of the child at adolescence								
Height *z*-score	0.06 ± 0.28	0.29 ± 0.07	0.50 ± 0.13	0.22	− 0.7 ± 0.3	− 0.1 ± 0.1	0.4 ± 0.1	< 0.01
Weight *z*-score	− 0.32 ± 0.31	0.20 ± 0.08	0.35 ± 0.15	0.14	0.2 ± 0.3	− 0.1 ± 0.1	0.4 ± 0.1	< 0.01
BMI, kg/m^2^	17.7 ± 0.9	19.2 ± 0.2	19.5 ± 0.4	0.22	21.5 ± 0.8	19.4 ± 0.2	20.4 ± 0.4	0.01
WC, cm	64.9 ± 2.5	68.8 ± 0.6	69.5 ± 1.2	0.26	70.4 ± 2.0	67.9 ± 0.5	70.6 ± 1.0	0.03
Fat mass index, kg/m^2^	2.2 ± 0.5	3.0 ± 0.1	2.9 ± 0.2	0.25	5.7 ± 0.5	4.5 ± 0.1	5.1 ± 0.2	0.01
Body fat percentage, %	12.4 ± 1.6	15.1 ± 0.4	14.4 ± 0.8	0.23	25.2 ± 1.5	22.8 ± 0.4	24.2 ± 0.7	0.10
Lean mass index, kg/m^2^	15.1 ± 0.5	15.8 ± 0.1	16.2 ± 0.2	0.10	15 ± 0.4	14.5 ± 0.1	14.9 ± 0	0.04
Overweight, *N* (%)	0 (0.0)	22 (14.6)	5 (11.4)	0.59	2 (20.0)	12 (7.2)	6 (14.3)	0.12
Sedentary behavior, *N* (%)								
< 2 h	7 (70.0)	100 (66.2)	21 (47.7)	0.10	3 (30.0)	93 (56.0)	23 (54.8)	0.33
2–3 h	1 (10.0)	28 (18.6)	16 (36.4)		3 (30.0)	47 (28.3)	13 (31.0)	
≥ 3 h	2 (20.0)	23 (15.2)	7 (15.9)		4 (40.0)	26 (15.7)	6 (14.2)	
Pubic hair appearance, *N* (%)								
Age < 12 years	3 (30.0)	42 (27.8)	11 (25.0)	0.92	9 (90.0)	108 (65.1)	28 (66.7)	0.31
Age ≥ 12 years	7 (70.0)	109 (72.2)	33 (75.0)		1 (10.0)	58 (34.9)	14 (33.3)	
RWG from 0 to 1.5 years, *N* (%)	7 (70.0)	51 (33.8)	10 (22.7)	0.02	9 (90.0)	65 (39.2)	10 (23.8)	< 0.01

Data are expressed as mean ± standard deviation or number (proportion in %)

*N* number, *SGA* small for gestational age, *AGA* appropriate for gestational age, *LGA* large for gestational age, *BMI* body mass index, *WC* waist circumference, *RWG* rapid weight gain

Table 2 shows the mean values of anthropometric and body composition indices according to postnatal weight gain (i.e., non-RWG and RWG). Gestational age was younger in the RWG group than in the non-RWG group. Offspring weight z-score at birth was lower in the RWG group than in the non-RWG group. There was a difference in pubic hair appearance in girls between the RWG and non-RWG groups. Parameters of body composition were larger in the RWG group than in the non-RWG group in both boys and girls.

**Table 2:** Characteristics of participants stratified by sex and rapid weight gain from birth to 1.5 years

	Boys			Girls		
	Non-RWG (*N* = 137)	RWG (*N* = 68)	*p* value	Non-RWG (*N* = 134)	RWG (*N* = 84)	*p* value
Maternal characteristics at pregnancy						
Age, years	29.7 ± 4.5	29.1 ± 4.7	0.35	29.1 ± 5.5	28.6 ± 4.6	0.52
Gestational age, weeks	39.2 ± 1.1	38.6 ± 1.1	< 0.01	39.2 ± 1.0	38.6 ± 1.1	< 0.01
Characteristics of child at birth						
Length *z*-score	0.30 ± 0.8	− 0.09 ± 0.89	< 0.01	0.34 ± 0.85	− 0.05 ± 0.93	< 0.01
Weight *z*-score	0.47 ± 1.0	− 0.16 ± 0.86	< 0.01	0.27 ± 0.79	− 0.47 ± 0.88	< 0.01
Characteristics of the child at age 1.5 years						
Height *z*-score	0.26 ± 0.8	0.82 ± 0.99	< 0.01	− 0.03 ± 0.99	0.65 ± 0.96	< 0.01
Weight *z*-score	0.05 ± 0.7	1.11 ± 0.92	< 0.01	− 0.03 ± 0.79	1.03 ± 1.03	< 0.01
Change in weight *z*-score from 0 to 1.5 years	− 0.42 ± 0.76	1.28 ± 0.46	< 0.01	− 0.30 ± 0.63	1.50 ± 0.69	< 0.01
Characteristics of the child at adolescence						
Height *z*-score	0.17 ± 0.86	0.62 ± 0.83	< 0.01	− 0.16 ± 0.91	0.19 ± 0.96	< 0.01
Weight *z*-score	0.02 ± 0.89	0.59 ± 1.06	< 0.01	− 0.20 ± 0.94	0.32 ± 0.86	< 0.01
BMI, kg/m^2^	18.8 ± 2.6	20.1 ± 3.2	< 0.01	19.2 ± 2.5	20.4 ± 2.8	< 0.01
WC, cm	67.5 ± 7.1	71.3 ± 9.0	< 0.01	67.6 ± 6.3	70.0 ± 6.8	< 0.01
Fat mass index, kg/m^2^	2.8 ± 1.5	3.4 ± 1.6	0.01	4.4 ± 1.5	5.1 ± 1.6	< 0.01
Body fat percentage, %	14.2 ± 5.0	16.1 ± 5.3	0.01	22.4 ± 4.8	24.3 ± 4.8	< 0.01
Lean mass index, kg/m^2^	15.6 ± 1.4	16.3 ± 1.9	< 0.01	14.4 ± 1.3	14.9 ± 1.4	< 0.01
Overweight, *N* (%)	12 (8.8)	15 (22.1)	0.01	9 (6.7)	11 (13.1)	0.15
Sedentary behavior, *N* (%)						
< 2 h	82 (59.9)	46 (67.6)	0.32	74 (55.2)	45 (53.6)	0.27
2–3 h	30 (21.9)	15 (22.1)		42 (31.4)	21 (25.0)	
≥ 3 h	25 (18.2)	7 (10.3)		18 (13.4)	18 (21.4)	
Pubic hair appearance, *N* (%)						
Age < 12 years	34 (24.8)	22 (32.4)	0.32	79 (59.0)	66 (78.6)	< 0.01
Age ≥ 12 years	103 (75.2)	46 (67.6)		55 (41.0)	18 (21.4)	
Size for gestational age, *N* (%)						
SGA	3 (2.2)	7 (10.3)	< 0.01	1 (0.7)	9 (10.7)	< 0.01
AGA	100 (73.0)	51 (75.0)		101 (75.4)	65 (77.4)	
LGA	34 (24.8)	10 (14.7)		32 (23.9)	10 (11.9)	

Data are expressed as mean ± standard deviation or number (proportion in %)

*N* number, *SGA* small for gestational age, *AGA* appropriate for gestational age, *LGA* large for gestational age, *BMI* body mass index, *WC* waist circumference, *RWG* rapid weight gain

Table 3 provides the adjusted means of BMI, WC, and body fat indices stratified by birthweight categories. After adjusting for potential confounding factors including RWG, there was a significant difference in the mean of FMI in girls and a marginally significant difference in boys.

**Table 3:** Least squares means and standard error of anthropometric variables and body fat at age 13–14 years according to birthweight for gestational age categories

	Birthweight for gestational age categories			*p* value for ANCOVA
	SGA	AGA	LGA	
Boys				
BMI	17.0 ± 1.1	19.1 ± 0.5	19.5 ± 0.7	0.06
WC	62.7 ± 2.9	68.5 ± 1.5	69.8 ± 1.9	0.06
Fat mass index	1.8 ± 0.6	3.1 ± 0.3	3.0 ± 0.4	0.06
Body fat percentage	10.8 ± 1.9	15.2 ± 1.0	14.8 ± 1.2	0.05
Girls				
BMI	21.0 ± 0.8	19.6 ± 0.2	20.7 ± 0.4	0.01
WC	69.4 ± 2.1	68.2 ± 0.5	71.2 ± 1.0	0.02
Fat mass index	5.4 ± 0.5	4.6 ± 0.1	5.3 ± 0.2	0.02
Body fat percentage	24.4 ± 1.5	23.0 ± 0.4	24.8 ± 0.8	0.08

*SGA* small for gestational age, *AGA* appropriate for gestational age, *LGA* large for gestational age, *BMI* body mass index, *WC* waist circumference, *ANCOVA* analysis of covariance

Adjusted for gestational age, sedentary behavior, and rapid weight gain in boys

Adjusted for maternal age at pregnancy and rapid weight gain in girls

Table 4 provides the adjusted means of BMI, WC, and body fat indices stratified by postnatal weight gain. After adjusting for potential confounding factors including birthweight categories, BMI, WC, and body fat indices were significantly larger in the RWG group than in the non-RWG group in both boys and girls.

**Table 4:** Least squares means and standard error of anthropometric variables and body fat at age 13–14 years according to weight gain status

	Non-RWG	RWG	*p* value for ANCOVA
Boys			
BMI	18.2 ± 0.4	19.7 ± 0.4	< 0.01
WC	66.0 ± 1.1	70.3 ± 1.2	< 0.01
Fat mass index	2.4 ± 0.2	3.1 ± 0.2	< 0.01
Body fat percentage	12.9 ± 0.7	15.2 ± 0.8	< 0.01
Girls			
BMI	19.8 ± 0.4	20.8 ± 0.4	0.01
WC	68.3 ± 0.9	70.5 ± 1.0	0.02
Fat mass index	4.7 ± 0.2	5.3 ± 0.2	0.01
Body fat percentage	22.9 ± 0.7	24.5 ± 0.7	0.03

*BMI* body mass index, *WC* waist circumference, *RWG* rapid weight gain, *ANCOVA* analysis of covariance

Adjusted for gestational age and birthweight for gestational age categories in boys

Adjusted for gestational age, pubic hair appearance, and birthweight for gestational age categories in girls

Table 5 provides standardized regression coefficients of birthweight categories and weight gain in early life for FMI in adolescents. There was a significant positive association between weight gain and FMI, but not between birthweight categories and FMI.

**Table 5:** Standardized regression coefficients of birthweight for gestational age categories and weight gain for fat mass index at age 13–14 years

Independent variables	Boys		Girls	
	Beta	*p* value	Beta	*p* value
Unadjusted				
Birthweight for gestational age categories	0.098	0.18	0.124	0.07
Change in weight gain from 0 to 1.5 years	0.217	< 0.01	0.279	< 0.01
Adjusted				
Birthweight for gestational age categories	0.115	0.13	0.133	0.06
Change in weight gain from 0 to 1.5 years	0.253	< 0.01	0.246	< 0.01

Adjusted for gestational age and sedentary behavior in boys

Adjusted for maternal age at pregnancy, gestational age, and pubic hair appearance in girls

Table 6 provides standardized regression coefficients of birthweight categories and change in BMI in early life for FMI in adolescents. There was a significant positive association between change in BMI and FMI.

**Table 6:** Standardized regression coefficients of birthweight for gestational age categories and change in BMI from 0 to 1.5 years for fat mass index at age 13–14 years

Independent variables	Boys		Girls	
	Beta	*p* value	Beta	*p* value
Unadjusted				
Birthweight for gestational age categories	0.279	0.22	0.339	0.14
Change in BMI from 0 to 1.5 years	0.245	< 0.01	0.250	< 0.01
Adjusted				
Birthweight for gestational age categories	0.294	0.20	0.390	0.09
Change in BMI from 0 to 1.5 years	0.259	< 0.01	0.216	< 0.01

Adjusted for gestational age and sedentary behavior in boys

Adjusted for maternal age at pregnancy, gestational age, and pubic hair appearance in girls

## Discussion

The present population-based retrospective cohort study examined associations of birthweight and RWG with DXA-measured body fat mass in school-aged students. We found that birthweight was not associated with fat mass in adolescents after adjusting for confounding factors including RWG. In contrast, RWG was significantly, positively associated with fat mass later in life, with a tendency for children with RWG to have an increased fat mass at age 13 to 14 years. Similarly, change in BMI in early life was positively associated with fat mass. These findings suggest that weight gain in early years has a greater impact on childhood fat mass than birthweight in children born at full-term. Given that growth status in early life has a greater impact on obesity in adolescents than birthweight, monitoring weight during infancy may help prevent obesity later in life.

Few studies have analyzed the effects of full-term birthweight and weight gain in early years on obesity in children. The FUPRECOL study found no significant differences in childhood BMI and WC between full-term SGA and full-term AGA [19]. However, that study did not investigate the potential modifying effects of weight gain in early years. The Generation R Study, which included about 4% preterm births in the study population, showed that skinfold thickness (subcutaneous fat thickness) did not significantly differ between children born SGA without RWG and those born AGA without RWG [9], but was larger in children born AGA with RWG compared to those born AGA without RWG [9]. In a prospective cohort study targeting 285 children with marginally low birth weight and 95 control children in Sweden, FMI at age 7 years was similar between children born SGA and AGA. The authors concluded that the magnitude of weight gain in early life predicted greater BMI and FMI at age 7 years [20]. These studies suggest that RWG, but not birthweight, is associated with BMI and fat mass in childhood [4, 9, 19, 20]. Our results showing that RWG early in life predicts obesity in childhood and adolescence are consistent with these findings.

Most children born SGA show catch-up growth (RWG), which is generally defined as a growth velocity (cm/year) greater than the median for chronological age and sex, within the first 2 years of life [21]. Children born SGA without catch-up growth may remain short-statured as adults [22]. Although catch-up growth is a common physiological process in children born SGA [23], it has been suggested to increase the risk of insulin resistance later in life [24]. Thus, monitoring weight changes in children born SGA is especially important.

Although the mechanism linking RWG and adolescent obesity remains unclear, several factors have been proposed for how RWG may lead to obesity. One factor may be the intrauterine and postnatal nutritional environment. For instance, one study reported that excessive gestational weight gain is associated with RWG in the early postnatal period [25]. Additional potential factors include formula feeding practices (e.g., adding cereal to the bottle, putting a baby to bed with the bottle, and overfeeding formula) and compositions (e.g., higher protein) which could contribute to RWG [26]. Epigenetic programming may also play a role [16, 27, 28].

On the other hand, an increasing number of reports on the topic of sedentary behavior have indicated that sedentary time is positively associated with overweight. A meta-analysis found a small but statistically significant positive relationship between TV viewing time and body fatness in youth [29]. However, one systematic review found insufficient evidence about the positive longitudinal relationship between sedentary time and fat mass [30]. Our results also showed no significant association between sedentary behavior and body fat in adolescent boys and girls (Table 3). The association between sedentary behavior and fat mass thus remains a matter of debate, and further studies will be needed to verify and build on our findings.

Strengths of the present study include the use of MCH Handbook records and DXA to measure body fat mass. Under the Maternal and Child Health Act in Japan, the MCH handbook is provided free of charge to expectant mothers who submit a notice of pregnancy to the government. The MCH handbook is a comprehensive record of prenatal checkups, delivery, child development, and vaccinations [13]. Therefore, data accuracy is expected to be high, minimizing recall bias.

This study also has some limitations. First, participants were selected from a few cities in Japan, and thus may not be representative of the entire Japanese population. Second, we could not control for all confounders, as data were unavailable for some items such as alcohol consumption, parity, socioeconomic status, passive smoking in childhood, breastfeeding, nutrition, physical activities, pubertal development, and environmental chemical exposure. A future study should include these potential confounders. Finally, the number of participants in the SGA category was small. Further studies with a large sample size will be needed to build upon the findings of the present study.

## Conclusion

Weight gain in early years has a greater impact on childhood fat mass than birthweight in children born at full-term. Our findings suggest that infants may be a target population for intervention to prevent obesity in children born at full-term. Monitoring weight during infancy may contribute to the prevention of adolescent obesity, a risk factor for cardiovascular disease.

## Data Availability

The datasets generated during the current study are not publicly available, but are available from the corresponding author on reasonable request.

## Abbreviations

AGA: Appropriate for gestational age
ANCOVA: Analysis of covariance
ANOVA: Analysis of variance
BMI: Body mass index
DXA: Dual-energy X-ray absorptiometry
MCH: Maternal and Child Health
JKB: Japan Kids Body-composition
LGA: Large for gestational age
RWG: Rapid weight gain
SGA: Small for gestational age
WC: Waist circumference

## References

[1] Kotsis V, Jordan J, Micic D, Finer N, Leitner DR, Toplak H, Tokgozoglu L, Athyros V, Elisaf M, Filippatos TD, Redon J, Redon P, Antza C, Tsioufis K, Grassi G, Seravalle G, Coca A, Sierra C, Lurbe E, Stabouli S, Jelakovic B, Nilsson PM (2018). Obesity and cardiovascular risk: a call for action from the European Society of Hypertension Working Group of Obesity, Diabetes and the High-risk Patient and European Association for the Study of Obesity: part A: mechanisms of obesity induced hypertension, diabetes and dyslipidemia and practice guidelines for treatment. J Hypertens.

[2] Reilly JJ, Kelly J (2011). Long-term impact of overweight and obesity in childhood and adolescence on morbidity and premature mortality in adulthood: systematic review. Int J Obes.

[3] Gaskins RB, LaGasse LL, Liu J, Shankaran S, Lester BM, Bada HS, Bauer C, Das A, Higgins R, Roberts M (2010). Small for gestational age and higher birth weight predict childhood obesity in preterm infants. Am J Perinatol.

[4] Ou-Yang MC, Sun Y, Liebowitz M, Chen CC, Fang ML, Dai W (2020). Accelerated weight gain, prematurity, and the risk of childhood obesity: A meta-analysis and systematic review. PLoS One.

[5] Lu Y, Pearce A, Li L (2020). Weight gain in early years and subsequent body mass index trajectories across birth weight groups: a prospective longitudinal study. Eur J Pub Health.

[6] Demerath EW, Reed D, Choh AC, Soloway L, Lee M, Czerwinski SA, Chumlea WC, Siervogel RM, Towne B (2009). Rapid postnatal weight gain and visceral adiposity in adulthood: the Fels Longitudinal Study. Obesity (Silver Spring).

[7] Fujita Y, Kouda K, Nakamura H, Iki M (2013). Association of rapid weight gain during early childhood with cardiovascular risk factors in Japanese adolescents. J Epidemiol.

[8] Spiegel E, Shoham-Vardi I, Sergienko R, Landau D, Sheiner E (2019). The association between birth weight at term and long-term endocrine morbidity of the offspring(). J Matern Fetal Neonatal Med.

[9] Taal HR, Vd Heijden AJ, Steegers EA, Hofman A, Jaddoe VW (2013). Small and large size for gestational age at birth, infant growth, and childhood overweight. Obesity (Silver Spring).

[10] Lei X, Zhao D, Huang L, Luo Z, Zhang J, Yu X, Zhang Y (2018). Childhood Health Outcomes in Term, Large-for-Gestational-Age Babies With Different Postnatal Growth Patterns. Am J Epidemiol.

[11] Laskey MA (1996). Dual-energy X-ray absorptiometry and body composition. Nutrition..

[12] Fujita Y, Kouda K, Ohara K, Nakamura H, Iki M (2020). Maternal pre-pregnancy underweight is associated with underweight and low bone mass in school-aged children. J Bone Miner Metab.

[13] Nakamura Y (2014). Maternal and Child Health: - Work together and learn together for maternal and child health handbook. Japan Med Assoc J.

[14] Itabashi K, Miura F, Uehara R, Nakamura Y (2014). New Japanese neonatal anthropometric charts for gestational age at birth. Pediatr Int.

[15] Suwa S, Tachibana K (1993). Standard Growth Charts for Height and Weight of Japanese Children from Birth to 17 Years Based on a Cross-sectional Survey of National Data. Clin Pediatr Endocrinol.

[16] Ong KK, Loos RJ (2006). Rapid infancy weight gain and subsequent obesity: systematic reviews and hopeful suggestions. Acta Paediatr.

[17] Cole TJ, Bellizzi MC, Flegal KM, Dietz WH (2000). Establishing a standard definition for child overweight and obesity worldwide: international survey. BMJ..

[18] Fujita Y, Kouda K, Ohara K, Nakamura H, Iki M (2019). Leptin mediates the relationship between fat mass and blood pressure: The Hamamatsu School-based health study. Medicine (Baltimore).

[19] Ramirez-Velez R, Correa-Bautista JE, Villa-Gonzalez E, Martinez-Torres J, Hackney AC, Garcia-Hermoso A (2017). Effects of preterm birth and fetal growth retardation on life-course cardiovascular risk factors among schoolchildren from Colombia: The FUPRECOL study. Early Hum Dev.

[20] Lindberg J, Norman M, Westrup B, Ohrman T, Domellof M, Berglund SK (2015). Overweight, Obesity, and Body Composition in 3.5- and 7-Year-Old Swedish Children Born with Marginally Low Birth Weight. J Pediatr.

[21] Houk CP, Lee PA (2012). Early diagnosis and treatment referral of children born small for gestational age without catch-up growth are critical for optimal growth outcomes. Int J Pediatr Endocrinol.

[22] Lee PA, Chernausek SD, Hokken-Koelega AC, Czernichow P (2003). International Small for Gestational Age Advisory Board consensus development conference statement: management of short children born small for gestational age, April 24-October 1, 2001. Pediatrics.

[23] van der Steen M, Hokken-Koelega AC (2016). Growth and Metabolism in Children Born Small for Gestational Age. Endocrinol Metab Clin N Am.

[24] Cho WK, Suh BK (2016). Catch-up growth and catch-up fat in children born small for gestational age. Kor J Pediatr.

[25] Subhan FB, Colman I, McCargar L, Bell RC, Team APS (2017). Higher Pre-pregnancy BMI and Excessive Gestational Weight Gain are Risk Factors for Rapid Weight Gain in Infants. Matern Child Health J.

[26] Appleton J, Russell CG, Laws R, Fowler C, Campbell K, Denney-Wilson E (2018). Infant formula feeding practices associated with rapid weight gain: A systematic review. Matern Child Nutr.

[27] Elks CE, Loos RJ, Sharp SJ, Langenberg C, Ring SM, Timpson NJ (2010). Genetic markers of adult obesity risk are associated with greater early infancy weight gain and growth. PLoS Med.

[28] Yeung MY (2006). Postnatal growth, neurodevelopment and altered adiposity after preterm birth--from a clinical nutrition perspective. Acta Paediatr.

[29] Marshall SJ, Biddle SJ, Gorely T, Cameron N, Murdey I (2004). Relationships between media use, body fatness and physical activity in children and youth: a meta-analysis. Int J Obes Relat Metab Disord.

[30] Chinapaw MJ, Proper KI, Brug J, van Mechelen W, Singh AS (2011). Relationship between young peoples’ sedentary behaviour and biomedical health indicators: a systematic review of prospective studies. Obes Rev.

